# Barley Yield Response to Nitrogen Application under Different Weather Conditions

**DOI:** 10.1038/s41598-019-44876-y

**Published:** 2019-06-11

**Authors:** Ryo Tanaka, Hiroshi Nakano

**Affiliations:** Kyushu Okinawa Agricultural Research Center, NARO, 496 Izumi, Chikugo, Fukuoka 833-0041 Japan

**Keywords:** Plant physiology, Heat

## Abstract

Barley, one of the most important crops worldwide, will be exposed to high air temperatures as a result of global warming. Since global warming is projected to progress with annual fluctuations, weather-adaptive cultivation techniques are needed in the area of barley production. This study aimed to determine the effect of nitrogen (N) application rate at heading on the grain yield of barley grown under different weather conditions based on two years of field experiments. Grain yield increased markedly with increasing N application rate in the 2017–2018 cropping season but not in the 2016–2017 cropping season. In contrast, late-emerging tillers clearly increased with increasing N application rate in the 2016–2017 cropping season but not in the 2017–2018 cropping season. Plants grown in the 2016–2017 cropping season produced relatively few grains due to the short period of tillering as a results of high air temperatures compared with those grown in the 2017–2018 crop season. Thus, in the 2016–2017 cropping season, N application could be used for the production of late-emerging tillers as a consequence of the limited sink capacity, whereas, in the 2017–2018 cropping season, it could be used effectively to increase grain yield.

## Introduction

Global average temperatures increased by 0.85 °C from 1880 to 2012^1^. By the end of this century, in the RCP2.6 scenario which predicts the estimated amount of CO_2_ emissions to be small, it will increase by 0.3–1.7 °C. On the other hand, in the more extreme RCP8.5 scenario, it will increase by 2.6–4.8 °C. Global warming has a negative impact on the grain yield of the important cereal crops such as maize, wheat, and barley^2^.

The world population is estimated to reach 9.8 billion people by 2050^3^. Global crop production must be increased substantially to feed such a large number of people. Since arable land for crop cultivation is limited worldwide^4^, improving crop yield per unit area is essential to resolve the global food issue.

Global production of barley is the fourth highest after that of maize, wheat, and rice^5^. Barley is used to malt for beer and for animal feed in the world. β-Glucan and flavonoids present in barley grain have received attention due to their biomedical effects^6,7^. In south western Japan, barley, which is generally sown in late November after harvesting rice in early October as a double-cropping system, is a very important crop and studies on using barley for whole-crop silage as well as for food are in progress^8^.

It has been reported that barley grain yield and grain weight decreased when plants ripened under high temperature^9–11^. A previous study found that N application at heading increased barley grain yield and grain weight^12^. Similar results were obtained in wheat in south western Japan^13,14^. However, another study showed that the application did not increase barley grain yield or grain weight^15^. Thus, the response of barley grain yield and grain weight may depend on environmental factors such as the year prevailing climate and site characteristics.

Global warming is considered to progress steadily, though with year-on-year fluctuations^16^. Hence, weather-adaptive cultivation techniques are needed over the crop production area. In the present study, we aimed to determine the effect of N application rate at heading on the grain yield of barley grown under different weather conditions before heading, by sowing the crop at three different sowing dates each year and over two years, to determine whether N application is a weather-adaptive technique to improve grain yield and possibly grain yield stability of barley over different weather conditions.

## Results

### Grain yield and its components in the 2016‒2017 crop season

**Figure 1:**
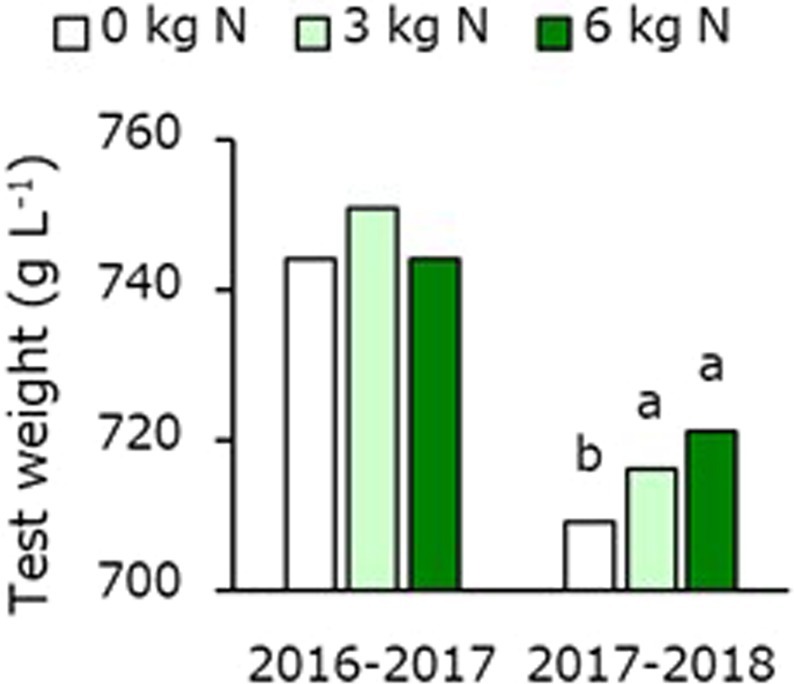
Mean test weight as affected by different nitrogen application rate at heading in the 2016–2017 and 2017–2018 crop seasons. Since there was no significant interaction between sowing date and nitrogen application rate at heading for test weight, each data represents mean value of all sowing times.

Grain yield was not affected by sowing date or N application rate at heading (Table 1). There was an interaction between sowing date and N application rate for the number of spikes m^−2^. In early sowings, spike number m^−2^ increased with increasing N application rate, whereas, in late sowings, it decreased with increasing N application rate. At 6 g m^−2^ N application, spike number m^−2^ decreased in response to delaying sowing date. At all N application rates, grain number per spike was the highest in normal sowings, followed by late and early sowings. An interaction also observed between sowing date and N application rate for 1000-grain weight. In late sowings, 1000-grain weight at 6 and 3 g m^−2^ N applications was greater than that at 0 g m^−2^ N application. At all N application rates, 1000-grain weight increased with delaying sowing date. Grain protein concentration increased as N application rate increase regardless of sowing date. Test weight was not affected by sowing date or N application rate (Fig. 1).

**Table 1:** Mean grain yield, its component, and protein concentration as affected by different sowing date and nitrogen application rate at heading in the 2016–2017 crop season.

Sowing date	Nitrogen rate	Grain yield	Grain number	Spike number	Grain number	1000-grain weight	Protein conc.
	(g m^−2^ N)	(g m^−2^)	(×10^3^ m^−2^)	(m^−2^)	(spike^−1^)	(g)	(%)
**Sowing date (S)**							
Early		483	10.5	642	16.4c	46.0c^†^	9.3
Normal		498	10.3	581	17.9b	48.1b	9.8
Late		543	11.0	564	19.6a	49.4a	9.8
**Nitrogen rate (N)**							
	0	506	10.7	586	18.2	47.4b	8.6c
	3	502	10.4	596	17.5	48.2a	9.7b
	6	517	10.8	605	18.1	47.9a	10.5a
**S × N**							
Early	0	476	10.3	597b	17.3	46.1B	8.2
	3	480	10.4	626b	16.7	46.1B	9.6
	6	494	10.8	703aA^‡^	15.3	45.8C	10.1
Normal	0	483	10.1	570	17.7	48.0A	8.9
	3	490	10.1	581	17.5	48.4A	9.9
	6	521	10.9	592B	18.4	48.0B	10.7
Late	0	558	11.5	591a	19.5	48.3bA	8.9
	3	537	10.7	581a	18.4	50.0aA	9.7
	6	537	10.7	520bB	20.7	50.0aA	10.9
**ANOVA**							
S		NS^§^	NS	NS	*	**	NS
N		NS	NS	NS	NS	**	***
S × N		NS	NS	*	NS	**	NS

*Significant at P < 0.05. **Significant at P < 0.01. ***Significant at P < 0.001. ^†^Means within a column followed by the same lowercase letter do not differ significantly (P < 0.05). ^‡^Means within a column followed by the same lowercase letter do not differ significantly (P < 0.05) among N application at heading for a given sowing date. Means within a column followed by the same uppercase letter do not differ significantly (P < 0.05) among sowing dates for a given N application at heading. ^§^Not significant at P < 0.05.

### Grain yield and its components in the 2017–2018 crop season

At all N application rates at heading, grain yield was higher in early and normal sowings than in late sowings (Table 2). At all sowing dates, grain yield enhanced with increasing N application rate. Grain number m^−2^ decreased in response to delaying sowing dates regardless of N application rate. This was because grain number per spike increased slightly but spike number m^−2^ decreased more markedly with later sowing dates. At all N application rates, 1000-grain weight was the greatest at normal sowing date, followed by late and early sowing dates. At all sowing dates, 1000-grain weight at 6 and 3 g m^−2^ N applications was greater than that at 0 g m^−2^ N application. Grain protein concentration increased as sowing date delayed regardless of N application rate and increased as N application rate increased regardless of sowing date. At all sowing dates, test weight increased with increasing N application rate (Fig. 1).

**Table 2:** Mean grain yield, its component, and protein concentration as affected by different sowing date and nitrogen application rate at heading in the 2017–2018 crop season.

Sowing date	Nitrogen rate	Grain yield	Grain number	Spike number	Grain number	1000-grain weight	Protein conc.
	(g m^−2^ N)	(g m^−2^)	(×10^3^ m^−2^)	(m^−2^)	(spike^−1^)	(g)	(%)
**Sowing date (S)**							
Early		675a^†^	14.8a	776a	19.1c	45.6c	9.6b
Normal		652a	13.3b	624b	21.3a	49.0a	9.7b
Late		571b	12.1c	595b	20.3b	47.3b	10.4a
**Nitrogen rate (N)**							
	0	612b	13.2	660	20.1	46.4b	8.8c
	3	637ab	13.4	669	20.2	47.5a	10.1b
	6	649a	13.5	666	20.5	48.0a	10.9a
**S × N**							
Early	0	669	14.9	778	19.2	44.9	8.5
	3	669	14.6	779	18.8	45.9	9.8
	6	688	15.0	773	19.5	45.9	10.6
Normal	0	632	13.1	610	21.5	48.2	8.5
	3	679	13.8	639	21.5	49.3	9.8
	6	646	13.0	624	21.0	49.6	10.9
Late	0	536	11.6	593	19.7	46.1	9.3
	3	563	11.9	591	20.2	47.3	10.7
	6	614	12.6	601	21.0	48.3	11.2
**ANOVA**							
S		***	***	**	*	**	**
N		*	NS	NS	NS	**	***
S × N		NS^‡^	NS	NS	NS	NS	NS

*Significant at P < 0.05. **Significant at P < 0.01. ***Significant at P < 0.001. ^†^Means within a column followed by the same lowercase letter do not differ significantly (P < 0.05). ^‡^Not significant at P < 0.05.

### Relationships between grain yield and its components

**Figure 2:**
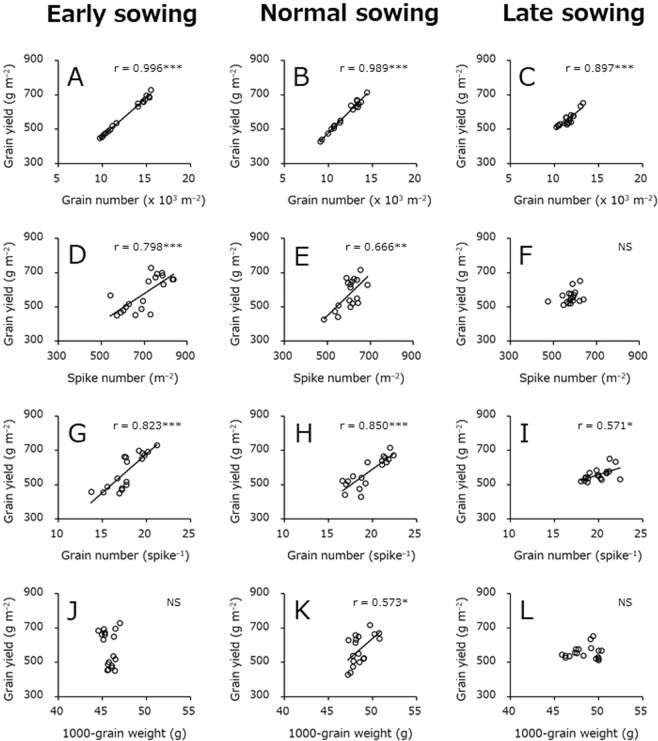
Relationships between grain yield and its components. Data obtained in the 2016–2017 and 2017–2018 crop seasons are included. (**A**–**C**) Represent the relationships between grain yield and grain number m^−2^ in early, normal, and late sowings, respectively. (**D**–**F**) Represent the relationships between grain yield and spike number m^−2^ in early, normal, and late sowings, respectively. (**G**–**I**) Represent the relationships between grain yield and grain number per spike in early, normal, and late sowings, respectively. (**J**–**L**) Represent the relationships between grain yield and 1000-grain weight in early, normal, and late sowings, respectively. *Significant at P < 0.05. **Significant at P < 0.01. ***Significant at P < 0.001. NS, not significant at P < 0.05.

Correlation coefficient analyses were conducted to determine the traits that were most closely associated with variation in grain yield (Fig. 2). At all sowing dates, grain number m^−2^ exhibited a strong positive correlation with grain yield. Spike number m^−2^ was positively correlated with grain yield in early and normal sowings but not in late sowing. Similarly, spike number m^−2^ and grain number per spike were positively related to grain yield regardless of sowing date. However, 1000-grain weight was positively related to grain yield only in normal sowing.

### Late tiller emergence

**Figure 3:**
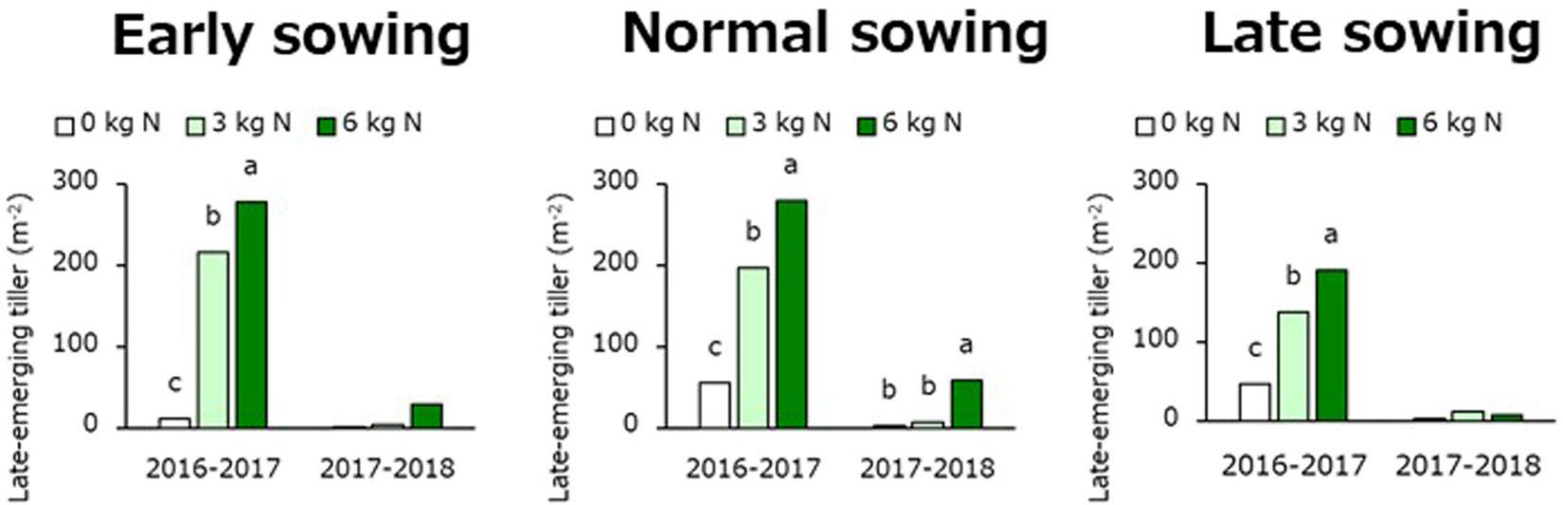
Mean late-emerging tiller number as affected by different nitrogen application rate at heading in the 2016–2017 and 2017–2018 crop season. There was a significant interaction between sowing date and nitrogen application rate at heading for late-emerging tiller number in the 2017–2018 crop season.

Late-emerging tiller increased with increasing N application rate at heading regardless sowing date in the 2016–2017 crop seasons (Fig. 3). However, late-emerging tiller increased with increasing N application rate in only normal sowing in the 2017–2018 crop season. In addition, there were more late-emerging tillers in the 2016–2017 crop season than there were in the 2017–2018 crop season.

### Relationships between growth-related traits and late tiller emergence

**Figure 4:**
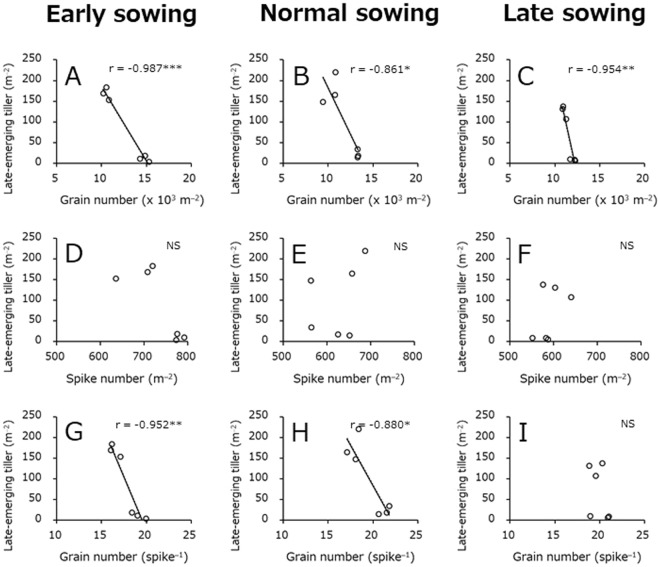
Relationships between late-emerging tiller number and grain number m^–2^, spike number m^–2^, and grain number per spike. Data obtained in the 2016–2017 and 2017–2018 crop seasons are included. (**A**–**C**) Represent the relationships between late-emerging tiller number and grain number m^−2^ in early, normal, and late sowings, respectively. (**D**–**F**) Represent the relationships between late-emerging tiller number and spike number m^−2^ in early, normal, and late sowings, respectively. (**G**–**I**) Represent the relationships between late-emerging tiller number and grain number per spike in early, normal, and late sowings, respectively. *Significant at P < 0.05. **Significant at P < 0.01. ***Significant at P < 0.001. NS, not significant at P < 0.05.

**Figure 5:**
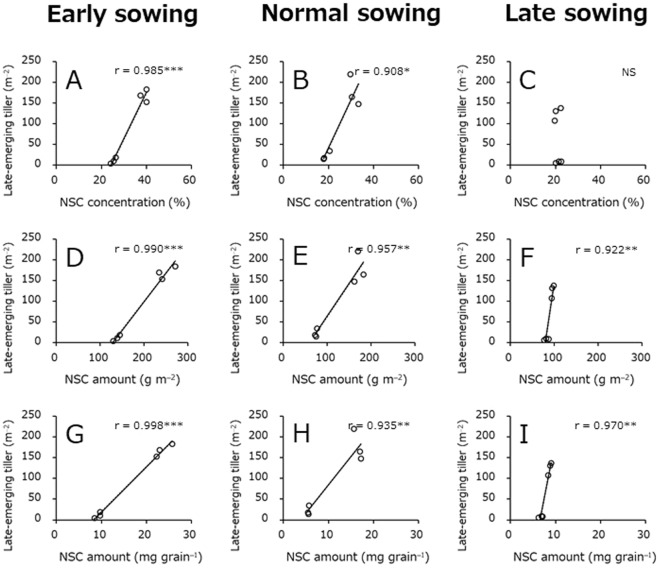
Relationships between late-emerging tiller number and NSC concentration, NSC amount m^−2^, and NSC amount per grain number at heading. Data obtained in the 2016–2017 and 2017–2018 crop seasons are included. (**A**–**C**) Represent the relationships between late-emerging tiller number and NSC concentration in early, normal, and late sowings, respectively. (**D**–**F**) Represent the relationships between late-emerging tiller number and NSC amount m^−2^ in early, normal, and late sowings, respectively. (**G**–**I**) Represent the relationships between late-emerging tiller number and NSC amount per grain at heading in early, normal, and late sowings, respectively. *Significant at P < 0.05. **Significant at P < 0.01. ***Significant at P < 0.001. NS, not significant at P < 0.05.

Correlation coefficient analyses were conducted to determine the traits that were most closely associated with variation in late tiller emergence (Figs 4 and 5). At the all sowing dates, late tiller emergence exhibited a strong negative correlation with grain number m^−2^ (Fig. 4) and a strong positive correlation with nonstructural carbohydrate (NSC) amount m^−2^ and NSC amount per grain number (Fig. 5).

#### Growth-related traits at heading in the 2016–2017 crop season

Although leaf sheath plus stem weight decreased with later sowing date, whole-plant weight was not affected by sowing time (Table 3). Leaf area index (LAI) was not affected by sowing time, while SPAD chlorophyll meter value was increased with delaying sowing date. NSC concentration, NSC amount m^−2^, and NSC amount per grain number decreased in response to delaying sowing date.

**Table 3:** Growth related traits at heading as affected by different sowing time in the 2016–2017 crop season.

Sowing date	Whole plant	Leaf blade	Dead leaf blade	Leaf sheath plus stem	Spike	LAI	SPAD value	NSC conc.	NSC amount	NSC amount
	(g m^−2^)							(%)	(g m^−2^)	(mg grain^−1^)
**Sowing date (S)**										
Early	899	154	26.0	633a^†^	86	5.39	41.5c	39.2a	249a	23.7a
Normal	778	122	13.8	551ab	91	4.53	46.0b	31.2b	171b	16.6b
Late	702	122	14.7	461b	104	4.99	51.2a	20.8c	96c	8.7c
**ANOVA**										
S	NS^‡^	NS	NS	*	NS	NS	**	***	***	***

*Significant at P < 0.05. **Significant at P < 0.01. ***Significant at P < 0.001. ^†^Means within a column followed by the same lowercase letter do not differ significantly (P < 0.05). ^‡^Not significant at P < 0.05.

#### Growth-related traits at heading in the 2017–2018 crop season

Whole- plant and leaf sheath plus stem weights decreased with delaying sowing date (Table 4). Dead leaf blade weight was the greatest in early sowing, followed by late and normal sowings. Spike weight increased in response to delaying sowing date. LAI was not affected by sowing date, while SPAD value was increased with delaying sowing date. NSC concentration and NSC amount m^−2^ were greater in early sowing than in normal and late sowings. NSC amount per grain number was the greatest in early sowing, followed by late and normal sowings.

**Table 4:** Growth related traits at heading as affected by different sowing time in the 2017–2018 crop season.

Sowing date	Whole plant	Leaf blade	Dead leaf blade	Leaf sheath plus stem	Spike	LAI	SPAD value	NSC conc.	NSC amount	NSC amount
	(g m^−2^)							(%)	(g m^−2^)	(mg grain^−1^)
**Sowing date (S)**										
Early	818a^†^	154	29.8a	540a	94b	6.14	42.7b	25.4a	137a	9.28a
Normal	652b	139	9.2c	394b	110a	5.94	47.0a	18.9b	74b	5.57c
Late	632b	128	13.3b	380b	111a	4.88	47.6a	21.4b	82b	6.77b
**ANOVA**										
S	**	NS^‡^	***	***	*	NS	***	*	***	**

*Significant at P < 0.05. **Significant at P < 0.01. ***Significant at P < 0.001. ^†^Means within a column followed by the same lowercase letter do not differ significantly (P < 0.05). ^‡^Not significant at P < 0.05.

#### Weather conditions

There were some differences marked in weather conditions between the 2016–2017 and 2017–2018 crop seasons (Table 5). Daily mean air temperatures during mid-November, late December, and early January in the 2016–2017 crop season were 2.8, 2.5, 2.2 °C higher, respectively, than those in an average season. However, daily mean air temperatures during early and mid-December, late January, and early February in the 2017–2018 crop season were 2.6, 2.7, 2.8, and 3.3 °C lower, respectively, than those in an average season. These data showed that the present field experiments in the 2016–2017 and 2017–2018 crop seasons were conducted under warm and cold winter conditions, respectively. In addition, daily mean air temperatures during March, April, and May in the 2017–2018 crop season, except for early May, were 1.0 to 2.1 °C higher than those in an average season.

**Table 5:** Mean daily air temperature, solar radiation, and precipitation at the Kyushu Okinawa Agricultural Research Center, NARO, Chikugo, Fukuoka, Japan, during the 2016–2017 and 2017–2018 crop seasons.

Month	Stage of month	Daily air temperature			Daily solar radiation			Daily precipitation		
		2016–7	2017–8	30-yr ave.^†^	2016–7	2017–8	30-yr ave.	2016–7	2017–8	30-yr ave.
		(°C)			(MJ m^−2^ d^−1^)			(mm d^−1^)		
November	Early	13.1	15.4	14.9	11.5	12.0	10.2	2.1	1.0	2.7
	Middle	15.5	10.9	12.7	8.2	10.1	9.1	6.9	0.2	2.6
	Late	11.3	9.8	10.6	7.3	7.3	8.5	3.4	1.0	2.4
December	Early	9.4	6.2	8.8	9.1	8.3	7.8	0.8	0.6	2.0
	Middle	8.2	4.6	7.3	7.7	6.7	7.5	2.1	0.0	1.5
	Late	8.8	5.5	6.3	6.6	7.6	7.7	4.1	0.5	1.2
January	Early	7.9	4.9	5.7	7.3	7.2	7.7	2.2	3.0	1.4
	Middle	4.6	4.9	5.5	9.7	7.9	7.6	0.1	2.2	2.0
	Late	4.8	2.3	5.1	11.0	9.9	8.6	0.6	1.1	1.9
February	Early	5.8	2.3	5.6	10.1	9.2	9.9	2.8	1.5	1.9
	Middle	6.5	5.0	6.7	13.2	10.4	10.6	2.2	0.9	2.8
	Late	7.0	7.6	7.8	12.0	12.1	11.4	1.8	3.6	3.1
March	Early	7.7	9.6	8.6	15.0	11.2	12.4	0.0	11.9	3.2
	Middle	9.4	11.9	9.9	14.6	12.4	13.3	2.2	6.6	3.8
	Late	10.2	12.6	11.3	11.4	18.9	14.1	1.9	2.8	3.9
April	Early	14.5	15.4	13.3	11.9	17.1	15.5	4.5	1.1	4.1
	Middle	16.5	16.5	15.0	17.0	17.9	16.2	0.7	5.1	4.4
	Late	17.0	18.2	16.6	20.5	18.2	17.0	0.6	8.4	5.4
May	Early	19.6	17.4	18.3	15.9	15.3	17.0	1.4	11.6	6.4
	Middle	19.7	21.2	19.5	21.3	19.0	17.2	6.2	3.2	6.5
	Late	22.2	22.6	20.8	22.5	16.7	17.5	0.3	0.7	4.0

^†^30-yr average (1989–2018) recorded at Kyushu Okinawa Agricultural Research Center, NARO for daily mean temperature and daily precipitation and the nearest weather station Saga for daily mean solar radiation.

Daily mean solar radiations during mid- and late January, mid-February, early March, late April, and mid- and late May in the 2016–2017 crop season and late March in the 2017–2018 crop season were 2.1, 2.4, 2.7, 2.6, 3.5, 4.1, 5.0, and 4.8 MJ M^−2^ d^−1^ higher than those in an average season. However, daily mean solar radiations during late March and early April in the 2016–2017 crop season were 2.7 and 3.6 MJ M^−2^ d^−1^ lower than those in an average season (Table 5).

Daily mean precipitations during mid-November in the 2016–2017 crop season and early March and early May in the 2017–2018 crop season were 4.3, 8.6, and 5.2 mm d^−1^ higher than those in an average season. However, daily mean precipitations during late April and early May were 4.8 and 5.0 mm d^−1^ in the 2016–2017 crop season lower than those in an average season.

#### Weather conditions at crop phenological growth stages

In all sowings, daily mean air temperatures at tillering were higher in the 2016–2017 crop season but lower in the 2017–2018 crop season than in an average season (Table 6). Daily mean air temperatures at ripening in both crop seasons were high compared with those in an average season regardless of sowing date and N application rate at heading.

**Table 6:** Mean daily air temperature, solar radiation, and precipitation at crop phenological growth stages in the 2016–2017 and 2017–2018 crop seasons.

Sowing date	Nitrogen rate	Daily air temperature			Daily solar radiation			Daily precipitation		
		Tillering^†^	Stem elongation^‡^	Ripening^§^	Tillering	Stem elongation	Ripening	Tillering	Stem elongation	Ripening
	(g m^−2^ N)	(°C)			(MJ m^−2^ d^−1^)			(mm d^−1^)		
**2016–2017 crop season**										
Early	0	9.7 (+1.1)^¶^	6.4 (−0.3)	13.8(+0.4)	8.0 (−0.1)	12.1 (+1.7)	15.1(−0.2)	3.0 (+1.2)	1.3 (−1.2)	1.9(−2.5)
	3			14.0(+0.4)			15.2(−0.1)			1.9(−2.6)
	6			14.3(+0.6)			15.3(−0.1)			1.8(−2.7)
Normal	0	7.1 (+0.5)	8.4 (−0.7)	16.5(+1.0)	9.1 (+0.7)	13.4 (+0.9)	16.0(−0.3)	2.0 (+0.2)	1.5 (−2.0)	1.9(−3.2)
	3			16.5(+0.9)			15.7(−0.6)			3.1(−2.0)
	6			16.6(+0.9)			15.9(−0.4)			3.1(−2.0)
Late	0	6.8 (+0.5)	9.9 (−0.8)	17.8(+1.1)	9.9 (+0.9)	13.8 (−0.1)	16.7(+0.1)	1.9 (−0.1)	1.7 (−2.1)	3.0(−2.5)
	3			17.9(+1.1)			16.9(+0.2)			2.9(−2.6)
	6			17.9(+1.1)			17.2(+0.5)			2.9(−2.7)
**2017–2018 crop season**										
Early	0	6.5 (−1.5)	7.3 (−0.6)	16.4(+1.8)	8.2 (0.0)	11.1 (−0.6)	18.5(+2.6)	1.1 (−0.8)	5.3 (+2.3)	4.3(−0.3)
	3			16.4(+1.7)			18.7(+2.7)			4.2(−0.5)
	6			16.4(+1.7)			18.7(+2.7)			4.2(−0.5)
Normal	0	5.4 (−1.4)	12.4 (+1.8)	17.0(+0.7)	8.8 (−0.1)	15.7 (+1.9)	17.2(+0.6)	2.2 (+0.1)	4.3 (+0.6)	6.9(+1.6)
	3			17.2(+0.8)			17.4(+0.8)			6.8(+1.4)
	6			17.3(+0.8)			17.4(+0.8)			6.6(+1.2)
Late	0	5.3 (−1.3)	13.5 (+1.9)	18.2(+1.1)	9.4 (0.0)	15.8 (+1.4)	17.4(+0.6)	2.6 (+0.5)	4.0 (0.0)	7.3(+1.6)
	3			18.2(+1.0)			17.5(+0.7)			7.1(+1.4)
	6			18.3(+1.0)			17.8(+0.9)			6.9(+1.2)

^†^Duration from sowing to jointing. ^‡^Duration from jointing to heading. ^§^Duration from heading to maturity. ^¶^Value in parenthesis indicate difference from 30-yr average.

In early sowings, daily mean solar radiations at stem elongation were higher in the 2016–2017 crop season than in an average season (Table 6). In normal and late sowings, daily mean solar radiations at stem elongation in the 2017–2018 crop season were high relative to those in an average season. In addition, in early sowings, daily mean solar radiations at ripening were higher in the 2017–2018 crop season than those in an average season.

In normal and late sowings, daily precipitations at stem elongation in the 2016–2017 crop season were lower than those in an average season. In early sowings, daily precipitations at stem elongation in the 2017–2018 crop season were higher than those in an average season. In addition, in all sowings, daily precipitations at ripening in the 2016–2017 crop season were lower than those in an average season.

#### Phenological development

Jointing in the 2016–2017 crop season occurred 8 to 18 days earlier than those in the 2017–2018 crop season did (Table 7). Heading date in the 2016–2017 crop season was 3 to 12 days earlier and the duration of stem elongation was 3 to 10 days longer than those in the 2017–2018 crop season.

**Table 7:** Phenological development in the 2016–2017 and 2017–2018 crop seasons.

Sowing date	Nitrogen rate	Sowing	Jointing	Heading	Maturity	Tillering^†^	Stem elongation^‡^	Ripening^§^
	(g m^−2^ N)	(m/d)				(day)		
**2016–2017 crop season**								
Early	0	11/7	1/18	3/12	5/2	72	53	51
	3				5/4			53
	6				5/6			55
Normal	0	11/25	2/18	3/28	5/11	85	38	44
	3				5/12			45
	6				5/13			46
Late	0	12/12	3/6	4/7	5/16	84	32	39
	3				5/17			40
	6				5/18			41
**2017–2018 crop season**								
Early	0	11/7	2/2	3/24	5/4	87	50	41
	3				5/5			42
	6				5/5			42
Normal	0	11/24	3/7	4/4	5/14	103	28	40
	3				5/15			41
	6				5/16			42
Late	0	12/12	3/14	4/10	5/18	92	27	38
	3				5/19			39
	6				5/20			40

^†^Duration from sowing to jointing. ^‡^Duration from jointing to heading. ^§^Duration from heading to maturity.

In both years, maturity was delayed a few days by the application of additional N at heading regardless of sowing date (Table 7).

## Discussion

As a result of global warming, barley crops will be exposed to higher air temperatures and their grain yields are estimated to decrease significantly^17^, which will exacerbate the problems associated with the continuing increase in the world population further^3^. To feed a large number of people, improved cultivation techniques to increase the grain yield of barley crops, one of the most important crops worldwide, are necessary. Here, we reveal the effect of N application rates at heading on grain yield of barley grown under different weather conditions before heading and propose the possible role of the weather-adaptive N application techniques to improve the grain yield of barley.

It has been indicated that barley grain yield and grain weight decreased when plants ripened under high temperatures^9–11^. A previous study has reported that N application at heading resulted in increasing grain yield and grain weight^12^, whereas another study has reported no such effect^15^. In the present study, grain yield and test weight increased markedly with increasing N application at heading in the 2017–2018 cropping season but not in the 2016–2017 cropping season regardless of sowing date (Tables 1 and 2). In contrast, late-emerging tillers clearly increased with increasing N application rate in the 2016–2017 cropping season but not in the 2017–2018 cropping season (Fig. 3). Below, we discuss the reasons why N application was effective at increasing yield in the 2017–2018 cropping season but not effective in the 2016–2017 cropping season.

At all sowing dates, grain number m^−2^, spike number m^−2^, and grain number per spike in the 2016–2017 cropping season were very low compared with those in the 2017–2018 cropping season (Tables 1 and 2). It has earlier been reported that these three yield components of barley decreased when plants were grown at high air temperatures at tillering as a consequence of a shortening of the duration of tillering^18,19^. Daily mean air temperatures in mid-November, late December, and early January in the 2016–2017 crop season were higher than those in average season, while daily mean air temperatures in early and mid-December and late January, and early February in the 2017–2018 crop season were lower than those in average season (Table 5). Consequently, at all sowing dates, daily mean air temperatures at tillering in the 2016–2017 crop season were higher than those in the 2017–2018 crop season (Table 6), a difference associated with a shorter tillering period in the 2016–2017 crop season, relative to that in the 2017–2018 crop season (Table 7). Meanwhile, it was reported that barley grain number per m^−2^, spike number, and grain number per spike decreased when plants grew under low solar radiation at stem elongation partially through a lack of photosynthetic products^20^. In normal and late sowings, daily mean solar radiations at stem elongation in the 2016–2017 crop season were lower than those in the 2017–2018 crop season (Table 6). However, in early sowings, the daily mean solar radiations at stem elongation in the 2016–2017 crop season were higher than those in the 2017–2018 crop season, although grain number m^−2^, spike number m^−2^, and grain number per spike in the 2016–2017 cropping season were very low compared with those in the 2017–2018 cropping season (Tables 1 and 2). In addition, a previous study indicated that barley yield was decreased by high precipitation during growth but not high temperature based on the results of multiple regression analysis among yield and weather conditions^21^. However, daily mean precipitations in the 2016–2017 crop season were much less than those in an average season (Table 5). These results suggested that low values for grain number per m^−2^, spike number, and grain number per spike in the 2016–2017 cropping season could be caused by high air temperatures at tillering.

Late-emerging tillers are considered to grow by using extra nutrients that cannot be translocated to the grain when its limited capacity. At all sowing dates and N application rates at heading, NSC concentration, NSC amount m^−2^, and NSC amount per grain number at heading in the 2016–2017 cropping season were clearly higher than those in the 2017–2018 cropping season (Tables 3 and 4). Furthermore, late-emerging tiller had strong negative and positive correlations with grain number m^−2^ and NSC amount, respectively, regardless of sowing date (Figs 4 and 5). Thus, the emergence of a large number of late-emerging tillers in the 2016–2017 cropping season might be caused by applying N to plants with a large amount of extra NSC due to the limited sink capacity.

For two-rowed barley, grains with a test weight ≥709 g L^−1^ and a whiteness ≥40 are highly evaluated in Japan^22^. In the present study, Grain protein concentration increased as sowing date delayed and N application rate increased (Tables 1 and 2). N application at heading increased test weight but also protein concentration (Tables 1 and 2 and Fig. 1). It was known that N application at heading is highly effective to increase grain protein concentration^13,14,23^. In addition, plants sown at late were increased protein concentration due to their high nitrogen concentration at heading in the 2017–2018 cropping season (Tables 2 and 4). Since a negative correlation between grain protein concentration and whiteness, one of the indexes of grain quality, has previously been indicated^10^, a large amount of N application is not recommended in late sowing in the production area.

In conclusion, N application at heading in the 2016–2017 crop season could be used for the production of late-emerging tillers, whereas that in the 2017–2018 crop season could be used more effectively to increase grain yield because of the adequate sink capacity. At all sowing dates, grain yield and test weight in the 2017–2018 crop season were markedly increased by N application when plants ripened under high air temperatures (Tables 2 and 6). Therefore, we can propose this weather-adaptive N application technique to improve grain yield and test weight. In particular, when high temperature is expected at ripening, N should not be applied to plants at heading grown under high air temperatures at tillering, whereas it should be applied to plants at heading grown under low air temperatures at tillering.

Grain number m^−2^ had the strongest positive correlation with grain yield regardless of sowing date (Fig. 2). This result was in close agreement with results from previous reports^24–26^. In high latitude region of Japan, future surface air temperature is predicted to particularly increase in winter rather than in summer^16,27^, suggesting that grain number m^−2^ may decrease in response to increasing air temperatures in winter. To facilitate steady and stable barley grain production under conditions of climate change, the development of techniques or varieties with the ability to produce sufficient grain numbers under high air temperature conditions in winter is needed.

## Materials and Methods

### Experimental design and crop management

The study was conducted in the 2016–2017 and 2017–2018 crop seasons on a fine-loamy, thermic Typic Endoaquept (a Lowland Paddy soil in the Japanese soil classification) at the Kyushu Okinawa Agricultural Research Center, NARO (33°12′N, 130°30′E), Chikugo, Fukuoka, Japan. The previous crops grown in the field of 2016−2017 and 2017−2018 crop seasons were rice and barley, respectively. Treatments included three sowing dates and three topdressings at heading, which were arranged as a split-plot experiment with three replicates in a randomised complete block design. The main plot and subplot were sowing date and N application rate at heading (i.e., when 90% of spikes have been headed), respectively.

The day before sowing, plots were manually supplied with 50 kg ha^−1^ N, 43 kg ha^−1^ P_2_O_5_, and 43 kg ha^−1^ K_2_O in the form of synthetic fertiliser in both crop seasons. The fertilisers were incorporated into the soil by ploughing. A two-rowed hulled barley variety ‘Haruka-Nijo’, which had recently been developed by Kyushu Okinawa Agricultural Research Center, NARO^28^ and has rapidly extended its acreage in southwestern Japan, was used. The seeds, which were wrapped with seeder tape, were drill-sown by hand at 158 seeds m^−2^ on 7 November (early), 25 November (normal), and 12 December (late) in 2016 and on 7 November (early), 24 November (normal), and 12 December (late) in 2017. Plants were manually supplied with 40 kg ha^−1^ N, 34 kg ha^−1^ P_2_O_5_, and 34 kg ha^−1^ K_2_O and 20 kg ha^−1^ N, 17 kg ha^−1^ P_2_O_5_, and 17 kg ha^−1^ K_2_O at Zadoks growth scale 14 (four leaves emerged) and 31 (first node detectable)^29^, respectively, in the form of synthetic fertiliser in both crop seasons. In addition, plants were manually supplied with 0, 30, or 60 kg ha^−1^ N in the form of ammonium sulfate at heading. After trimming, each plot was 4.7 m wide × 2.8 m long and 6.8 m wide × 2.8 m long in the 2016–2017 and 2017–2018 crop seasons, respectively, with one ridge containing four rows spaced at 30 cm.

### Sampling and measurement

At heading, plants from 1.12 m^2^ (1.4 m wide × 0.8 m long) were sampled from each plot in both crop seasons. The number of spikes was counted and approximately 10% of the plants were separated into green leaf blades, dead leaf blades, leaf sheaths, leaf sheath plus stems, and spikes. After the area of green leaf blades was measured with a leaf area metre (LI-3050A/4, Li-COR, Inc., Lincoln, NE, USA), each plant part was dried at 80 °C in a ventilated oven for 2 d along with the remining plants to determine dry weight. The dried samples of leaf sheath plus stem were ground to a powder with a vibrating sample mill (TI-1001, CMT Co., Ltd., Tokyo, Japan) to measure NSC concentrations. The concentrations of NSC were determined as described by Ohnishi and Horie^30^.

At maturity, plants from 2.52 m^2^ (1.4 m wide × 1.8 m long) were harvested in both crop seasons and air-dried until they reached a constant weight. Late emerging spikes were separated. The number of spikes was counted and the plants were threshed to determine their grain weight. The grains with a thickness ≥2.5 mm were recorded for grain weight. The number of grains with a thickness ≥2.5 mm required to make up 40 g was counted with a multi auto counter (KC-1M5, Fujiwara Scientific Co., Ltd., Tokyo, Japan) and the 1000-grain weight was calculated from this value. The test weight was measured with an instant multiple moisture tester (PM830-2, Kett Electric Laboratory Co., Ltd., Tokyo, Japan). Grain yield and 1000-grain weight were corrected to a 12.5% moisture concentration basis. The grain protein concentration was determined by Kjeldahl method (N × 5.83). In addition, the number of late-emerging spikes were counted.

### Statistical analysis

Statistical analyses were performed using a general linear model in SAS Add-In for Microsoft Office (version 7.13 HF4, SAS Institute, Cary, NC, USA). Analysis of variance (ANOVA) was used to test the effect of sowing date and N application rate at heading on yield, its components, and growth-related traits. Year, replication × year, and sowing date × replication × year were considered to be random effects. There were significant interactions between sowing date × year and among sowing date × N application rate × year for grain yield; therefore, ANOVA was conducted separately each year. Replication and sowing date × replication were considered to be random effects. Significant treatment effects (*P* < 0.05) were explored using Fisher’s protected least significant difference (LSD).
